# Cyclin D1 expression predicts postoperative distant metastasis and survival in resectable esophageal squamous cell carcinoma

**DOI:** 10.18632/oncotarget.9078

**Published:** 2016-04-28

**Authors:** Xue Hou, Run-Bin Liang, Jin-Chang Wei, Ying Xu, Jian-Hua Fu, Rong-Zhen Luo, Jie-Hua He, Lan-Jun Zhang, Peng Lin, Hao-Xian Yang

**Affiliations:** ^1^ Department of Medical Oncology, Sun Yat-sen University Cancer Center, State Key Laboratory of Oncology in South China, Collaborative Innovation Center for Cancer Medicine, Guangzhou City, Guangdong Province, China; ^2^ Department of Thoracic Surgery, Sun Yat-sen University Cancer Center, State Key Laboratory of Oncology in South China, Collaborative Innovation Center for Cancer Medicine, Guangzhou City, Guangdong Province, China; ^3^ Guangdong Esophageal Cancer Institute, Guangzhou City, Guangdong Province, China; ^4^ Department of Thoracic Surgery, Linzhou Esophageal Cancer Hospital, Yaocun Town, Linzhou City, Henan Province, China; ^5^ Institute of Medical Statistics and Epidemiology, Sun Yat-sen University Medical College, Guangzhou City, Guangdong Province, China; ^6^ Department of Pathology, Sun Yat-sen University Cancer Center, State Key Laboratory of Oncology in South China, Collaborative Innovation Center for Cancer Medicine, Guangzhou City, Guangdong Province, China

**Keywords:** esophageal neoplasm, esophageal squamous cell carcinoma, cyclin D1, surgery, metastasis

## Abstract

**Purpose:**

We aim to identify esophageal squamous cell carcinoma patients with increased risk of postoperative metastases.

**Results:**

A high level of cyclin D1 expression, together with poor tumor cell differentiation and advanced tumor stages, increased risk of postoperative metastasis and decreased distant metastasis-free survival in ESCC in both cohorts. A high level of cyclin D1 expression also decreased overall survival in the training cohort (*p* < 0.01) but not in the validation cohort (*p* = 0.415). However, when the two cohorts of patients were pooled to obtain a larger case number, a high level of cyclin D1 expression was again demonstrated as an independent predictor that decreased overall survival (*p* < 0.01).

**Methods:**

We used data from two institutions to establish training (*n* = 319) and validation (*n* = 164) cohorts. Tissue microarrays were generated for immunohistochemical evaluation. The correlation among cyclin D1 expression, clinicopathologic variables, postoperative distant metastases, overall survival, and distant metastasis-free survival were analyzed. Multivariate analyses were used to test the independent factors impacting postoperative distant metastases and survival. The outcomes generated from the training cohort were then tested using the validation cohort and pooled dataset.

**Conclusions:**

High level of cyclin D1 expression increased distant metastasis, decreased overall survival and distant metastasis-free survival in resectable ESCC. Using a combination of cyclin D1 expression, tumor cell differentiation grade, and tumor stages, identifying patients with increased risk of postoperative metastases becomes possible.

## INTRODUCTION

Squamous cell carcinoma is the most common pathological type of esophageal cancer in the Asia [1]. Surgery is the most promising curative therapy for operable esophageal squamous cell carcinoma (ESCC); however, postoperative distant metastasis is still the primary cause of death among these patients, and the five-year overall survival (OS) is only approximately 40% [2–4]. Once the distant organ metastasis occurred, the five-year survival rate is only approximately 7% [5]. Therefore, it is essential to select patients with a high risk of postoperative distant metastases and administer them intensive surveillance and individualized adjuvant therapy to improve long-term outcomes.

In current clinical practice, physicians assess the prognosis of ESCC patients mainly based on the American Joint Committee on Cancer (AJCC) staging system. However, the postoperative recurrence of individual ESCC, even with the same AJCC stage, may vary considerably. The genetic heterogeneity of the tumors may contribute to these discrepancies. Being one of the cyclin-dependent kinases, cyclin D1 is an important cell cycle regulator and is amplified in a variety of cancers, including ESCC [6]. However, the correlation between cyclin D1 expression and prognosis of ESCC patients is still controversial [7–20] and requires further investigation.

In this study, we investigated the correlation of cyclin D1 expression with postoperative distant metastases, overall survival (OS), and distant metastasis-free survival (DMFS) in resectable ESCC patients using one dataset for training and another independent dataset for validation.

## RESULTS

### General patient characteristics

As a result, 319 cases and 164 cases fit the inclusion criteria and established the training cohort and validation cohort, respectively (Table 1). The proportion of male patients was higher in the training cohort than the validation cohort. The tumor length was longer in the validation cohort than the training cohort. There were more patients with long tumor length, advanced pathological T categories, and advanced pathological AJCC stages in the validation cohort than the training cohort. The age distribution, tumor location, surgical approaches, pathological nodal categories, and cyclin D1 expression were well balanced between the two patient cohorts. The number of patients with high risk and low risk of distant organ metastasis was 94 (29.5%) and 225 (70.5%), respectively in the training cohort; the corresponding number in the validation cohort was 76 (46.3%) and 88 (53.7%), respectively, with a statistically significant difference between the two cohorts (*p* < 0.01).

**Table 1:** Clinicopatholigic characteristics of the two cohorts of patients

Variables	Training cohort (*n* = 319)	Validation cohort (*n* = 164)	*p*
	Patient No. (%)	Patient No. (%)	
Cyclin D1 expression			
Low	183 (57.4)	89 (54.3)	0.516
High	136 (42.6)	75 (45.7)	
Sex			
Male	240 (75.2)	107 (65.2)	0.021
Female	79 (24.8)	57 (34.8)	
Age (years, mean ± sd)	55.9 ± 9.4	57.0 ± 7.3	0.195
Tumor location			
Upper third	48 (15.0)	26 (15.9)	0.826
Middle third	151 (47.3)	81 (49.4)	
Lower third	120 (37.6)	57 (34.8)	
Surgical approaches			
Left thoracotomy	238 (74.6)	114 (69.5)	0.334
Ivor-Lewis	13 (4.1)	11 (6.7)	
Cervico-thoraco-abdominal	68 (21.3)	39 (23.8)	
Tumor length (cm)	4.3 ± 1.8	4.8 ± 2.2	0.026
Cell differentiation			
Well	81 (25.4)	26 (15.9)	< 0.01
Moderate	165 (51.7)	108 (65.9)	
Poor	73 (22.9)	30 (18.3)	
Pathological T category			
T1	29 (9.1)	9 (5.5)	< 0.01
T2	113 (35.4)	9 (5.5)	
T3	175 (54.9)	139 (84.8)	
T4	2 (0.6)	7 (4.3)	
Pathological N category			
N0	217 (68.0)	120 (73.2)	0.374
N1	71 (22.3)	26 (15.9)	
N2	26 (8.2)	14 (8.5)	
N3	5 (1.6)	4 (2.4)	
AJCC stage			
I	110 (34.5)	15 (9.1)	< 0.01
II	130 (40.8)	101 (61.6)	
III	79 (24.8)	48 (29.3)	
Total	319 (100)	164 (100)	

sd, standard deviation; AJCC, American Joint Committee on Cancer.

The median time from surgery to the last time of contact for the training cohort and validation cohort was 10.7 years (range: 7.8 to 15.7 years) and 9.9 years (range: 5.5 to 12.7 years), respectively. The major metastatic sites for both the training cohort and validation cohort were the lung (41.5% vs. 36.5%), liver (17.0% vs. 19.7%), and bone (16.0% vs. 17.1%).

### Cyclin D1 expression in the training cohort

**Figure 1:**
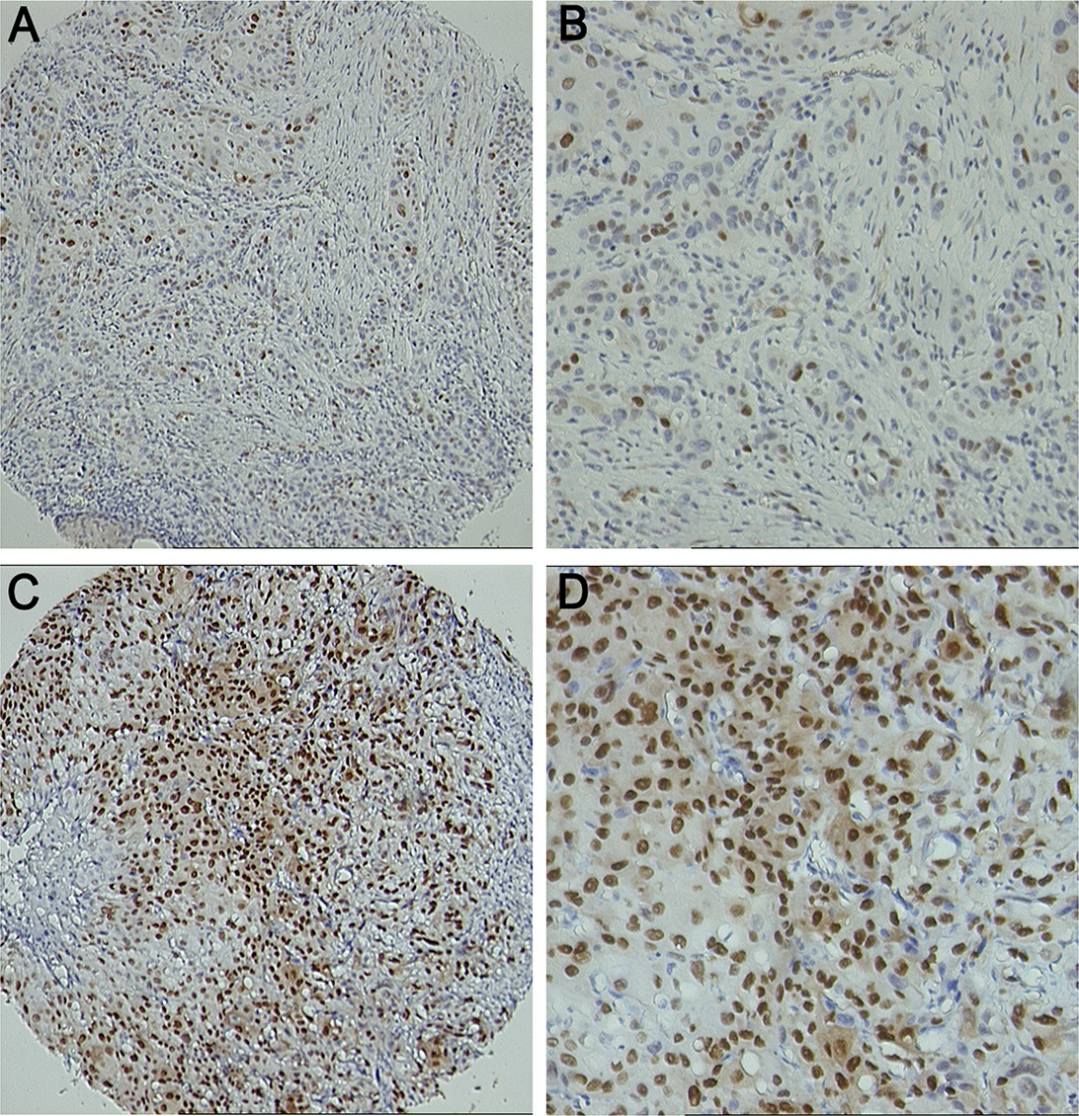
Representative figures of IHC staining for cyclin D1 expression (**A**) 100× for weak cyclin D1 staining; (**B**) 200× for weak cyclin D1 staining; (**C**) 100× for strong cyclin D1 staining; (**D**) 200× for strong cyclin D1 staining.

The cyclin D1 was mainly stained in the nuclei of the tumor cells (Figure 1). According to the receiver operating characteristic (ROC) analysis, the immunoreactivity score (IRS) value closest to the point of both maximum sensitivity and specificity in predicting distant metastasis was 3.33. Therefore, we defined 3.33 as the cutoff value of a high or low level of cyclin D1 expression. In patients with a low level of cyclinD1 expression, the relative risk for postoperative distant metastasis was 0.60 (95% confidence interval [CI]: 0.43 to 0.84, *p* < 0.01), indicating that a low level of cyclin D1 expression decreased distant metastasis in the training cohort. The level of cyclin D1 expression was comparable between the two cohorts of ESCC specimens (*p* = 0.516, Table 1).

Detailed information for cyclin D1 expression and clinicopathologic variables in the training cohort is provided in Table S1. Logistic regression analyses for the training cohort demonstrated that a high level of cyclin D1 expression, together with male sex, elderly age, poor tumor cell differentiation, and advanced AJCC stages, were independent factors that increased postoperative distant metastases (Table 2).

**Table 2:** Logistic regression models of the two cohorts of patients (dependent variable = distant metastasis in five years after surgery)

Prognostic Variables	Training Cohort (n = 319)				Validation Cohort (n = 164)			
	OR	95% CI for OR		*p*	OR	95% CI for OR		*p*
		Lower	Upper			Lower	Upper	
Gender (female vs. male)	0.251	0.113	0.559	< 0.01	0.771	0.341	1.742	0.531
Age (> 60 years vs. ≤ 60 years)	1.810	1.001	3.274	0.050	0.991	0.416	2.364	0.984
Tumor location	0.870	0.573	1.322	0.515	0.922	0.544	1.564	0.765
Tumor length (> 2.5 cm vs. ≤ 2.5 cm)	1.476	0.605	3.597	0.392	22.291	2.782	178.628	< 0.01
Cell differentiation	2.400	1.561	3.690	< 0.01	2.574	1.234	5.369	0.012
AJCC stage	3.093	2.053	4.661	< 0.01	4.200	1.967	8.970	< 0.01
Cyclin D1 (High level vs. Low level)	1.988	1.128	3.505	0.017	2.713	1.230	5.983	0.013

CI, confidence interval; OR, odds ratio; AJCC, the American Joint Committee on Cancer.

### Cyclin D1 expression in the validation cohort

When the cutoff value of 3.33 was applied in the validation cohort, 45.7% (75/164) of ESCC specimens were observed to have high levels of cyclin D1 expression. In patients with IRS ≤ 3.33, the relative risk for postoperative distant metastasis was 0.613 (95% CI: 0.438 to 0.858, *p* < 0.01).

Detailed information for cyclin D1 expression and clinicopathologic variables in the validation cohort is described in Table S1. Similar to that in the training cohort, logistic regression analyses for the validation cohort demonstrated that a high level of cyclin D1 expression, together with long tumor length, poor tumor cell differentiation, and advanced AJCC stages, were independent factors increased postoperative distant metastases (Table 2).

### Cyclin D1 expression in the pooled cohort

To enlarge the case number to guarantee more powerful statistics, we pooled the training cohort and validation cohort for further analyses. As predicted, the logistic regression analysis using the pooled data confirmed that a high level of cyclin D1 expression, male sex, long tumor length, poor tumor cell differentiation, and advanced AJCC staging were independent factors that increased postoperative distant metastases (Table S2).

### Survival

**Figure 2:**
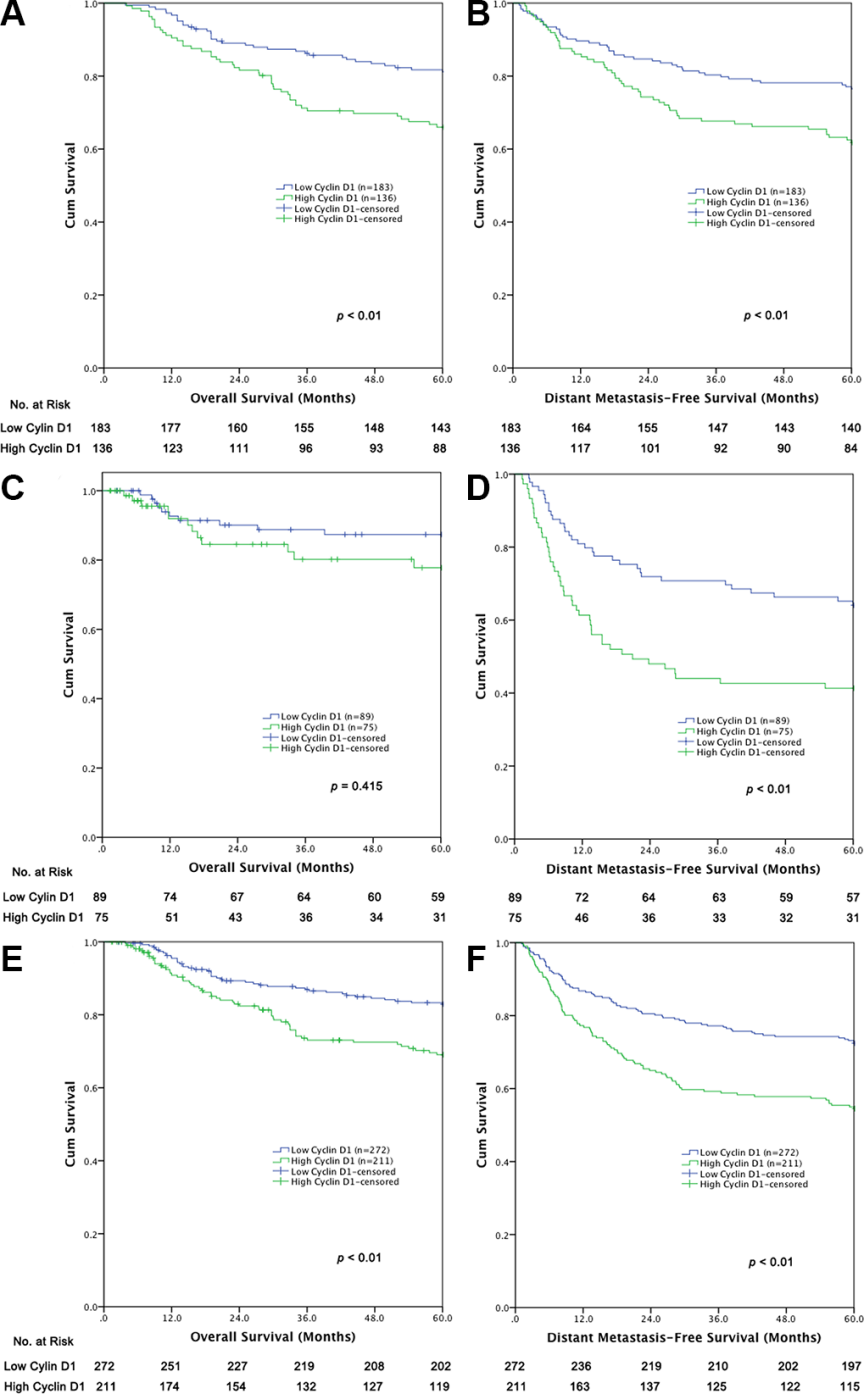
Survival curves of (A) OS in the training cohort (*n* = 319), (B) DMFS in the training cohort (*n* = 319), (C) OS in the validation cohort (*n* = 164), (D) DMFS in the validation cohort (*n* = 164), (E) OS in the pooled cohort (*n* = 483), (F) DMFS in the pooled cohort (*n* = 483)

The patients with a high level of cyclin D1 expression exhibited a decreased survival time, compared to patients with a low level of cyclin D1 expression in terms of both OS and DMFS in the training cohort (Figure 2A, 2B). A similar DMFS difference was also observed in the validation cohort (Figure 2D), but the OS difference was not statistically significant (Figure 2C). A multivariate Cox regression analysis confirmed that a high level of cyclin D1 was an independent factor decreasing both the OS and DMFS in the training cohort (Table S3, S4). In the validation cohort, a multivariate Cox regression analysis demonstrated that high cyclin D1 expression level decreased DMFS (Table S4), but not OS (Table S3).

We propose that the small case number may account for the statistically insignificant OS difference between the two groups of the cyclin D1 expression level in the validation cohort. Therefore, we pooled the training cohort and validation cohort together to enlarge the case number in a further analysis. As predicted, a statistically significant difference was observed in terms of both OS and DMFS between the patients with high and low levels of cyclin D1 expression in the pooled dataset. The multivariate Cox regression analysis confirmed that a low level of cyclin D1 expression, together with short tumor length, well tumor cell differentiation, and earlier AJCC staging, is an independent predictor favoring both OS and DMFS in the pooled cohort (Table 3).

**Table 3:** Prognostic factors for OS and DMFS by multivariate Cox regression analysis for pooled cohort of patients (*n* = 483)

Prognostic Variables	OS (n = 483)				DMFS (n = 483)			
	HR	95% CI for HR		*p*	HR	95% CI for HR		*p*
		Lower	Upper			Lower	Upper	
Gender (female vs. male)	0.600	0.374	0.963	0.034	0.730	0.508	1.049	0.089
Age (> 60 years vs. ≤ 60 years)	1.588	1.091	2.311	0.016	1.185	0.868	1.616	0.285
Tumor location	1.000	0.767	1.305	0.997	0.970	0.784	1.201	0.780
Tumor length (> 2.5 cm vs. ≤ 2.5 cm)	2.082	1.041	4.163	0.038	2.947	1.542	5.630	< 0.01
Cell differentiation	1.758	1.307	2.365	< 0.01	1.738	1.370	2.203	< 0.01
AJCC stage	2.160	1.628	2.865	< 0.01	2.358	1.867	2.979	< 0.01
Cyclin D1 (High level vs. Low level)	1.728	1.188	2.512	< 0.01	1.678	1.239	2.272	< 0.01

OS, overall survival; DMFS, distant metastasis-free survival; CI, confidence interval; HR, hazard ratio; AJCC, the American Joint Committee on Cancer.

## DISCUSSION

Our data demonstrated that a high level of cyclin D1 expression, together with poor tumor cell differentiation and advanced AJCC stage, predicted high risk of postoperative distant metastases in operable ESCC. As predicted, long-term survival also decreased in patients with a high level of cyclin D1 expression, opposed to patients with a low level. These outcomes were generated by a large volume of cases in the training cohort and confirmed by the independent validation cohort.

According to the practice guidelines of the National Comprehensive Cancer Network (NCCN), adjuvant therapy is not recommended for completely resected ESCC [21]. However, a previous study demonstrated that more than 40% of completely resected cases developed tumor recurrence [4]. Therefore, identifying the patients with a high risk of postoperative distant organ metastasis and providing tailored adjuvant therapy may improve long-term outcomes of resected ESCC. Our data indicated that in clinical practice, resectable ESCC patients with high levels of cyclin D1 expression, poor cell differentiation, and advanced pathological AJCC staging could be at high risk of distant metastases, thus close follow-up is needed or adjuvant therapy might be recommended. Conversely, patients with low levels of cyclin D1 expression, well cell differentiation, and early pathological AJCC staging predicts low risk of postoperative distant metastasis and routine surveillance is preferred. In this study, we used immunohistochemical (IHC) techniques that are already widely used conveniently and affordably in laboratories. This approach makes our result more adaptable to clinical practice.

Cyclin D1 is an important protein for the G1-S cell cycle phase transition that participates in cell proliferation and differentiation [22], and has been demonstrated to promote the progression of several human tumor types, including esophageal cancer [6, 23]. However, the effect of cyclin D1 on the prognosis of ESCC is still controversial. Several studies suggested that cyclin D1 amplification or over-expression decreased OS in ESCC [11–20], while others not [7–10]. Nevertheless, the case numbers in most of the previous studies were small, and only one of them was larger than 180 [12]. The small case numbers may partially contribute to these discrepancies. Data of 416 cases from the Research Committee on Malignancy of Esophageal Cancer, Japanese Society for Esophageal Diseases showed that increased cyclin D1 expression was a significant prognostic factor that decreased OS in patients with ESCC [12]. However, logistic regression analysis of 368 cases in that study indicated that cyclin D1 expression tended to increase the risk of hematogenous recurrence in the node-positive patients, but the *p* value is not statistically significant (*p* = 0.072) [12]. With an increased case number, similar outcomes from the dataset of the Japanese Society for Esophageal Disease might be achieved with that of our dataset. The majority of previous studies only considered OS, while studies concerning both OS and the recurrence-free survival were rare [17, 20]. Furthermore, to our knowledge, no previous published studies used postoperative distant metastasis as the primary endpoint.

To our knowledge, this study is the largest series focusing on cyclin D1 expression and prognosis of ESCC reported to date. Our data showed that there are some differences in clinicopathologic characteristics between the two cohorts of patients. Nevertheless, cyclin D1 increased postoperative distant metastasis could still be confirmed by the validation cohort, suggesting that this outcome is repeatable with good representativeness and potential for broad application. Besides, the prognostic value of cyclin D1 in patients' OS and DMFS was also determined in this study, which supplies more comprehensive outcomes. The large number of cases, multivariate analyses, and validation of results at another independent institution make our results more reliable, reproducible, and representative.

The limitations of this study must also be considered. First, some variation in methodological factors for IHC, such as different primary antibodies, wide range of dilutions, and the relatively difficult process of scoring for IHC staining, may contribute to different results of protein expression and therefore hinder its application in clinical practice. Second, the local recurrence rate was not determined in this study due to the respective nature. Further studies based on prospectively collected data are warranted.

In conclusion, a high level of cyclin D1 expression, together with poor tumor cell differentiation and advanced AJCC stage, increased risk of postoperative distant metastasis and decreased survival in patients with resectable ESCC. Therefore, it can help to identify patients with high risk of postoperative metastases.

## MATERIALS AND METHODS

### Patient selection

This study was approved by the Ethics Committee of the Sun Yat-sen University Cancer Center. We used two independent cohorts of patients to establish the training cohort (from Sun Yat-sen University Cancer Center in South China) and validation cohort (from Linzhou Esophageal Cancer Hospital in North China), respectively. Both centers have broad experience in esophageal surgery. The databases of the two cohorts of patients had been described previously [24]. The training cohort data came from an esophageal cancer database for consecutive surgery from January 1997 to January 2004. We used data from the training cohort to generate a cutoff value for cyclin D1 expression to divide the patients into groups of both a high level and low level of cyclin D1 expression. We then used the validation cohort to test the efficacy of the cutoff value in predicting postoperative distant organ metastases and survival. The validation cohort data were from an esophageal cancer database of patients who received surgical treatment for curative purposes between August 2000 and June 2007. The seventh edition of the AJCC cancer staging system was employed to re-stage the cases [25].

The inclusion criteria had been previously described [24]: (1) the disease was histologically defined as ESCC; (2) the patient underwent complete resection; (3) there was complete information for stage grouping; (4) the disease was pathological AJCC stages I–III; (5) the resections were neither preceded nor followed by adjuvant chemotherapy or radiotherapy; (6) for patients who were recorded with distant organ metastasis during follow-up, the metastatic organs were clearly recorded; and (7) there were adequate paraffin-embedded cancer tissue samples for use in constructing the tissue microarray. Patients with a history of concurrent malignant disease or other previous primary cancers and any operative deaths were excluded from this study.

Distant metastasis was defined as recurrence in other tissue or organs beyond the surgical field and lymph nodes. The high risk of postoperative distant metastasis was defined as distant metastasis that occurred within five years after surgery. The low risk of postoperative distant metastasis was defined as no postoperative distant metastasis within five years after a minimum follow-up of five years.

### Patient follow-up

In general, a follow-up examination was conducted every three months for the first year, every four months for the second year, and then twice a year thereafter. The routine examination during follow-up included a physical examination, blood chemistry, measurement of serum tumor markers, X-ray or computerized tomography (CT) scan of the chest, esophagography, ultrasonography or CT scan for the upper abdomen, and endoscopy. If tumor recurrence was suspected, then a biopsy was recommended if it was considered necessary for pathological confirmation. If the patient had specific symptoms, then the examination was performed as soon as possible. The patient survival time was measured from the date of surgery to the date of the event or last follow-up.

### Tissue microarrays immunohistochemical (IHC) staining and quantification

We prepared tissue microarrays for IHC staining to determine uniform and simultaneous protein expression in multiple tissue samples. For each patient sample, three tissue cylinders were taken from different representative tumor regions to generate the tissue microarrays [24]. The rabbit cyclin D1 monoclonal antibody (RM-9104-S0, 1:100, Neomarkers) was used as the first antibody for IHC staining, and the detailed methods for tissue microarray construction, IHC staining, and scoring were described elsewhere [24]. Two independent observers (RZ Luo and JH He) blinded to the clinicopathological information determined the immunoreactivity score (IRS) for cyclin D1 staining. The staining results were scored based on the following criteria: (a) percentage of positive tumor cells in the tumor tissue: zero (0%), one (1%–10%), two (11%–25%), three (26%–50%), four (51%–75%), or five (76%–100%); and (b) signal intensity: zero (no staining), one (weak staining), two (moderate staining), or three (strong staining) [24, 26–28]. The IRS was calculated by multiplying the score for the percentage of positive cells by the intensity score (range of 0 to 15) [24, 26–28]. If the conclusion was still controversial after the specimens were rescored, then a third pathologist intervened and worked collaboratively to reach a consensus. The average IRS of each core determined by the two pathologists was assigned as the staining result for the core. The average IRS of three cores for each case was assigned as the final staining result of the patient.

### Statistical analysis

The SPSS statistical software package (Standard version 16.0; IBM, Chicago, IL, USA) was used for data analysis. The mean values were presented as the mean ± standard deviation (SD). Independent *t*-tests were used to compare groups of continuous, normally distributed variables. The Pearson chi-square test was used to determine the significance of differences between groups for dichotomous variables. All statistical tests were two-tailed, and *p* < 0.05 was considered statistically significant.

To avoid a predetermined cutoff value, a receiver operating characteristic (ROC) analysis was used to define the cutoff value of IRS for cyclin D1 expression in the training cohort. The ROC analysis was performed by the MedCalc statistical software package 11.0.1 (MedCalc Software bvba, Mariakerke, Belgium). The score closest to the point of both maximum sensitivity and specificity was selected as the cutoff point associated with the greatest number of cases correctly classified as having or not having the clinical outcome. A logistic regression analysis was used to determine the independent factors associated with a high risk of distant metastasis. The Cox regression model was used to determine the independent factors impacting OS and DMFS. The cutoff value of IRS for cyclin D1 expression generated by the training cohort was then used in the validation cohort and the pooled dataset to test its ability to predict postoperative distant organ metastasis and survival.

## SUPPLEMENTARY MATERIALS TABLES


