# Supplementary material for: Cyclin D1 expression predicts postoperative distant metastasis and survival in resectable esophageal squamous cell carcinoma
Source: Oncotarget. 2016 Apr 28;7(21):31088–96. doi: 10.18632/oncotarget.9078 (PMC5058741; doi:10.18632/oncotarget.9078)
Supplement: Supplementary file 1 [file oncotarget-07-31088-s001.pdf]

Cyclin D1 expression predicts postoperative distant metastasis and survival in resectable esophageal squamous cell carcinoma

Supplementary Materials

Supplementary Table S1: Cyclin D1 expression and clinicopathologic variables

Variables	Training Cohort			Validation Cohort		
	Low	High	*p*	Low	High	*p*
Sex						
Male	139	101	0.729	58	49	0.982
Female	44	35		31	26	
Age						
≤ 60	123	83	0.253	72	46	< 0.01
> 60	60	53		17	29	
Cell differentiation						
Well	53	28	0.059	16	10	0.355
Moderate	96	69		60	48	
Poor	34	39		13	17	
Pathological T category						
T1	18	11	0.811[a]	4	5	0.893[a]
T2	68	45		5	4	
T3	96	79		77	62	
T4	1	1		3	4	
Pathological N category						
N0	129	88	0.623[a]	65	55	0.762[a]
N1	39	32		16	10	
N2	13	13		6	8	
N3	2	3		2	2	
AJCC stage						
I	70	40	0.259	8	7	0.926
II	70	60		56	45	
III	43	36		25	23	
Risk of distant metastasis						
Low	141	84	< 0.01	57	31	< 0.01
High	42	52		32	44	

AJCC: American Joint Committee on Cancer; [a]Fisher's exact test.

Supplementary Table S2: Logistic regression model of pooled dataset (*n* = 483; dependent variable = distant metastasis in five years after surgery)

Characteristics	OR	95% CI for OR		*p*
		Lower	Upper	
Gender (female vs male)	0.507	0.302	0.851	0.010
Age (> 60 years vs ≤ 60 years)	1.427	0.889	2.292	0.141
Tumor location	0.928	0.675	1.276	0.646
Tumor length (> 2.5 cm vs ≤ 2.5 cm)	3.459	1.586	7.543	< 0.01
Cell differentiation	2.362	1.654	3.374	< 0.01
AJCC stage	3.392	2.397	4.799	< 0.01
Cyclin D1 (High level vs Low level)	2.219	1.421	3.466	< 0.01

OR: odds ratio; CI: confidence interval; AJCC: American Joint Committee on Cancer.

Supplementry Table S3: Prognostic factors for OS by multivariate Cox regression analysis for both cohorts of patients

Prognostic Variables	Training Cohort (*n* = 319)				Validation Cohort (*n* = 164)			
	HR	95% CI for HR		*p*	HR	95% CI for HR		*p*
		Lower	Upper			Lower	Upper	
Gender (female vs. male)	0.508	0.282	0.914	0.024	0.890	0.369	2.142	0.794
Age (> 60 years vs. ≤ 60 years)	1.537	1.007	2.346	0.046	1.574	0.630	3.932	0.332
Tumor location	0.991	0.731	1.342	0.952	1.010	0.575	1.772	0.973
Tumor length (> 2.5 cm vs. ≤ 2.5 cm)	1.837	0.832	4.060	0.133	2.635	0.607	11.439	0.196
Cell differentiation	1.933	1.405	2.660	< 0.01	0.810	0.362	1.809	0.606
AJCC stage	2.041	1.515	2.749	< 0.01	3.307	1.522	7.185	< 0.01
Cyclin D1 (High level vs. Low level)	1.864	1.213	2.862	< 0.01	1.250	0.566	2.758	0.581

OS: overall survival; CI: confidence interval; HR: hazard ratio; AJCC: the American Joint Committee on Cancer.

Supplementary Table S4: Prognostic factors for DMFS by multivariate Cox regression analysis for both cohorts of patients

Prognostic Variables	Training Cohort (n = 319)				Validation Cohort (n = 164)			
	HR	95% CI for HR		p	HR	95% CI for HR		p
		Lower	Upper			Lower	Upper	
Gender (female vs. male)	0.459	0.256	0.825	< 0.01	0.876	0.536	1.431	0.597
Age (> 60 years vs. ≤ 60 years)	1.405	0.937	2.108	0.100	1.077	0.634	1.829	0.783
Tumor location	0.946	0.707	1.267	0.712	1.014	0.739	1.393	0.929
Tumor length (> 2.5 cm vs. ≤ 2.5 cm)	1.707	0.810	3.600	0.160	7.616	1.851	31.331	< 0.01
Cell differentiation	1.790	1.326	2.418	< 0.01	1.646	1.084	2.498	0.019
AJCC stage	2.172	1.629	2.897	< 0.01	2.651	1.694	4.149	< 0.01
Cyclin D1 (High level vs. Low level)	1.608	1.071	2.415	0.022	1.795	1.139	2.828	0.012

DMFS: distant metastasis-free survival; CI: confidence interval; HR: hazard ratio; AJCC: the American Joint Committee on Cancer.
